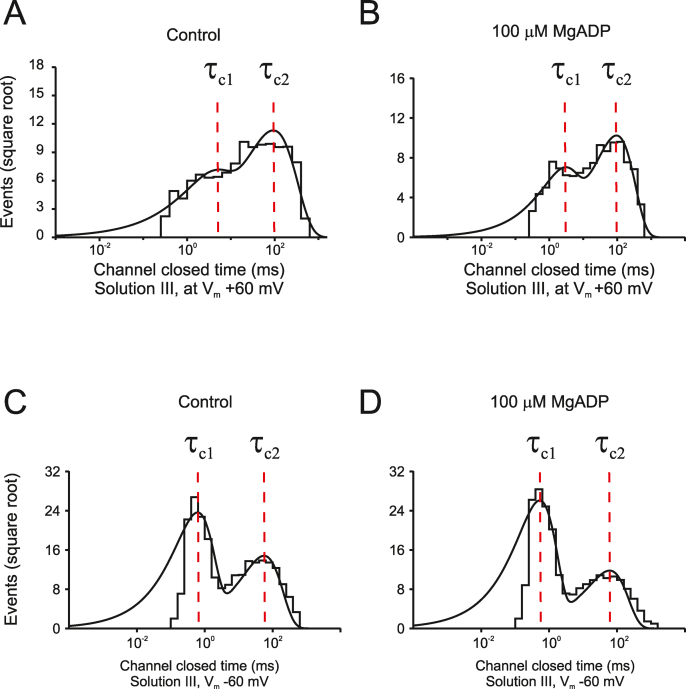# Inward and outward currents of native and cloned K(ATP) channels (Kir6.2/SUR1) share single-channel kinetic properties

**DOI:** 10.1016/j.bbrep.2022.101260

**Published:** 2022-04-08

**Authors:** Robert Bränström, Erik Berglund, Robin Fröbom, Ingo B. Leibiger, Barbara Leibiger, Craig A. Aspinwall, Olof Larsson, Per-Olof Berggren

**Affiliations:** a The Rolf Luft Research Center for Diabetes and Endocrinology, Sweden; b Endocrine and Sarcoma Surgery Unit, Department of Molecular Medicine and Surgery, Karolinska Institutet, Karolinska University Hospital, Stockholm, Sweden; c Department of Chemistry and Biochemistry, University of Arizona, Tucson, AZ, USA

**Keywords:** ATP-sensitive K^+^ (K(ATP)) channel, β-cells, Single channel kinetics, K(ATP), ATP-sensitive potassium channel, Kir, Inward rectifier K^+^ channel, SUR, subunit sulfonylurea receptor

## Abstract

**Background:**

The ATP-sensitive K^+^ (K(ATP)) channel is found in a variety of tissues extending from the heart and vascular smooth muscles to the endocrine pancreas and brain. Common to all K(ATP) channels is the pore-forming subunit Kir6.x, a member of the family of small inwardly rectifying K^+^ channels, and the regulatory subunit sulfonylurea receptor (SURx). In insulin secreting β-cells in the endocrine part of the pancreas, where the channel is best studied, the K(ATP) channel consists of Kir6.2 and SUR1. Under physiological conditions, the K(ATP) channel current flow is outward at membrane potentials more positive than the K^+^ equilibrium potential around −80 mV. However, K(ATP) channel kinetics have been extensively investigated for inward currents and the single-channel kinetic model is based on this type of recording, whereas only a limited amount of work has focused on outward current kinetics.

**Methods:**

We have estimated the kinetic properties of both native and cloned K(ATP) channels under varying ionic gradients and membrane potentials using the patch-clamp technique.

**Results:**

Analyses of outward currents in K(ATP) and cloned Kir6.2ΔC26 channels, alone or co-expressed with SUR1, show openings that are not grouped in bursts as seen for inward currents. Burst duration for inward current corresponds well to open time for outward current.

**Conclusions:**

Outward K(ATP) channel currents are not grouped in bursts regardless of membrane potential, and channel open time for outward currents corresponds to burst duration for inward currents.

## Highlights

• Outward K(ATP) channel currents are not grouped in bursts regardless of membrane potential.
• No differences in the number of kinetic states could be seen for single channels between native K(ATP) channels, Kir6.2ΔC26 alone and co-expressed with SUR1 for outward currents.
• K(ATP) channel open time for outward currents corresponds to burst duration for inward currents.

## 1 Introduction

The ATP-sensitive K^+^ (K(ATP)) channel couples metabolism to electrical activity [1,2]. In pancreatic β-cells, where the physiological action is best understood, the K(ATP) channel closes in response to an increased ATP/ADP ratio. Closure of K(ATP) channels depolarizes the plasma membrane, which opens voltage-gated Ca^2+^ channels, resulting in increased cytosolic levels of Ca^2+^ that trigger exocytosis [2,3]. The K(ATP) channel consists of the pore-forming subunit Kir6.x, which belongs to the family of small inwardly rectifying K^+^ channels, and the regulatory subunit sulfonylurea receptor (SURx). In heart and smooth muscle Kir6.1 (encoded by *KCNJ8*) and Kir6.2 (gene code *KCNJ11*), together with SUR2 (*ABCC9*) constitute the active K(ATP) channels, whereas in the neuroendocrine cells such as the endocrine pancreatic β-cell, the K(ATP) channel consists of Kir6.2 and SUR1 (*ABCC8*) [4,5].

Kinetic properties of the K(ATP) channel, both native and cloned, have been extensively characterized regarding inward currents by using symmetrical K^+^ gradients across the membrane [[6], [7], [8]]. High extracellular K^+^ concentrations elicit K(ATP) channel activity in both cell-attached and inside-out patches at physiological membrane potentials (V_m_), approximately −60 mV. In this configuration, K^+^ current flux through the channel is inward, and single-channel kinetic analyses have demonstrated that channel openings are grouped in bursts, with mean open and closed times within the burst in the low ms range and burst durations of 5–20 ms [[6], [7], [8], [9]]. Under theses conditions the kinetic model is best fitted to a four-state linear kinetic scheme [7,8]. However, at physiological ionic gradients ([K^+^]_o_ ∼5 mM and [K^+^]_i_ ∼155 mM) current flows outward at membrane potentials positive to the K^+^ equilibrium potential (−78 mV). The kinetic pattern under these conditions has not been as fully analyzed, although it stands clear that openings are much slower and not grouped in bursts [9,10]. In this study we have investigated the K(ATP) channel kinetics for inward and outward currents in the presence and absence of known modulators of kinetic patterns, e.g. MgADP [10].

## 2 Materials and methods

### 2.1 Animals and preparation of cells

Electrophysiological studies of the native K(ATP) channel were performed on excised patches from β-cells isolated from adult, male and female, obese mice (*ob/ob*). The animals were obtained from a local colony and were fasted for 24-h before decapitation. Islets were isolated using a collagenase technique [11]. Collagenase was obtained from Boehringer Mannheim (GmbH, Germany). A cell suspension was prepared as previously described [12]. The cells were maintained for 1–3 days in RPMI 1640 culture medium (Flow Laboratories, Scotland, UK), containing 11 mM glucose, supplemented with 10% fetal bovine serum and antibiotics (100 IU/mL penicillin, 100 μg/mL streptomycin, and 60 μg/mL gentamycin). The cells were seeded into Petri dishes (Corning Glass Works, Corning, NY) and incubated at 37 °C and 5% CO_2_.

### 2.2 Xenopus oocytes

Oocytes were collected from extra-large *Xenopus laevis* females. The animals were anaesthetized with 1.5 g 3-aminobenzoic acid methyl ester per L of water (Sigma, St. Louis, MO, USA). Oocytes were removed via a small abdominal incision and were defolliculated using a collagenase A method, described elsewhere [13]. Oocytes, stage V-VI, were injected with 0.5–5 ng of mRNA/50 nL sterile RNase-free water encoding Kir6.2ΔC26, or Kir6.2ΔC26+SUR1. Oocytes were maintained at 19 °C for 2–5 days before use.

### 2.3 Ethics and animal use

All animal handling complied with EU Directive 2010/63/EU for animal experiments, and all experiments were approved by the Ethics Committee.

### 2.4 mRNA preparation

The generation of pB.mKir6.2ΔC26 and pB.SUR1 plasmids was described previously [13]. All vector constructs were verified by DNA sequence analysis. Plasmid DNA was prepared using a QIAprep Spin Miniprep Kit (Qiagen GmbH, Hilden, Germany) and purified using a GenePrep Kit (Ambion, Austin, TX, USA). The respective plasmid DNA was linearized by digestion with *Xba*I, purified by phenolchloroform treatment, and ethanol-precipitated. The DNA pellet was re-dissolved in water and an aliquot containing 0.5–1 μg DNA was used for *in vitro* transcription. Capped mRNA was synthesized by employing the mMESSENGER mMACHINE T7 Kit (Ambion). The purified mRNA was dissolved in 10 mM Tris-HCl (pH 7.4) and stored in aliquots at −80 °C.

### 2.5 Electrophysiology

Recordings were obtained using the patch-clamp technique [14] and an Axopatch 200 amplifier (Axon Instrument, CA, USA). During the experiment the current signal was stored on magnetic tape using a VCR (Sony-200, Sony, Tokyo, Japan). Channel currents were recorded with pipette solutions containing (in mM): 138 NaCl, 5.5 KCl, 1.2 MgCl_2_, 2.6 CaCl_2,_ and 5 HEPES-NaOH (solution I), or 143 NaCl, 0.5 KCl, 1.2 MgCl_2_, 2.6 CaCl_2_ and 5 HEPES-NaOH (solution II), or 150 KCl, 1.2 MgCl_2_, 2.6 CaCl_2_ and 5 HEPES-KOH (solution III). In solution I, II and III pH was adjusted to 7.40. Intracellular (bath) solution consisted of (in mM): 120 KCl, 1.0 MgCl_2_, 10 EGTA, 25 KOH, and 5 HEPES-KOH at pH 7.15. ADP (Sigma) was added as Na ^+^ salt and Mg^2+^ was added to maintain an excess of Mg^2+^. All reagents were of analytical grade. To study K(ATP) channel kinetic properties, inside-out patches were excised from the cells. Pipettes were pulled from borosilicate glass (Hilgenberg, Malsfeld, Germany) and coated with Sylgard (Dow Corning, Kanagawa, Japan) near the tips to reduce electrical noise. Electrodes had resistances between 3 and 5 MΩ. Channel records are displayed according to the convention that upward deflections denote outward currents, and *vice versa*. The experiments were performed at room temperature, 20–22 °C.

### 2.6 Data analysis

Records were filtered at 2 kHz (−3 dB value, 8-pole Bessel filter, Frequency Devices, Haverhill, MA, USA), digitized at 10 kHz using an Axon instrument analogue digital converter (TL-1). Digitized segments of current records (20 s) were used to determine single-channel activity and kinetics using pCLAMP version 6.0 software (Axon Instrument). Before analysis, the digitized segments were enhanced using a cubic spline interpolation program to a final sampling frequency of 20 kHz. The analysis of channel open and closed time was restricted to segments of the experimental records containing a maximum of one active channel, and within 10 min from the patch is excised. Open time histograms are displayed using a linear graph, whereas channel closed time are displayed using a logarithmic axis (channel closed time histograms can be found in supplemental figures). By using the method of maximum likelihood [16], the kinetic constants (τ_*j*_) were derived by approximation of the data to probability density functions (pdf):

$$\text{F}\left(\text{t}\right)=\overset{m}{\underset{j=1}{\sum }}\left(\frac{a_{j}}{\tau_{j}}\right)e^{-t/\tau_{j}}$$

where *a*_*j*_ represents the relative area of the component. The numbers of fitted components were compared statistically using the log likelihood ratio. Channel bursts were determined as the sum of event lengths ended by a closed event longer than the burst length delimiter (τ_cutoff_). Determining the true burst length is not a trivial exercise and several methods are available. τ_cutoff_ was estimated by plotting the number of events per burst *versus* τ_cutoff_, which has been used previously [15] (Fig. 4A). Data are presented as means ± SEM, and *n* in text and figure legend represent number of patch-clamp recordings. Effects on channel activity were compared using Student's *t*-test (paired and unpaired), and *P* values < 0.05 were considered significant, whereas *n.s.* denotes not significant.

**Fig. 1:**
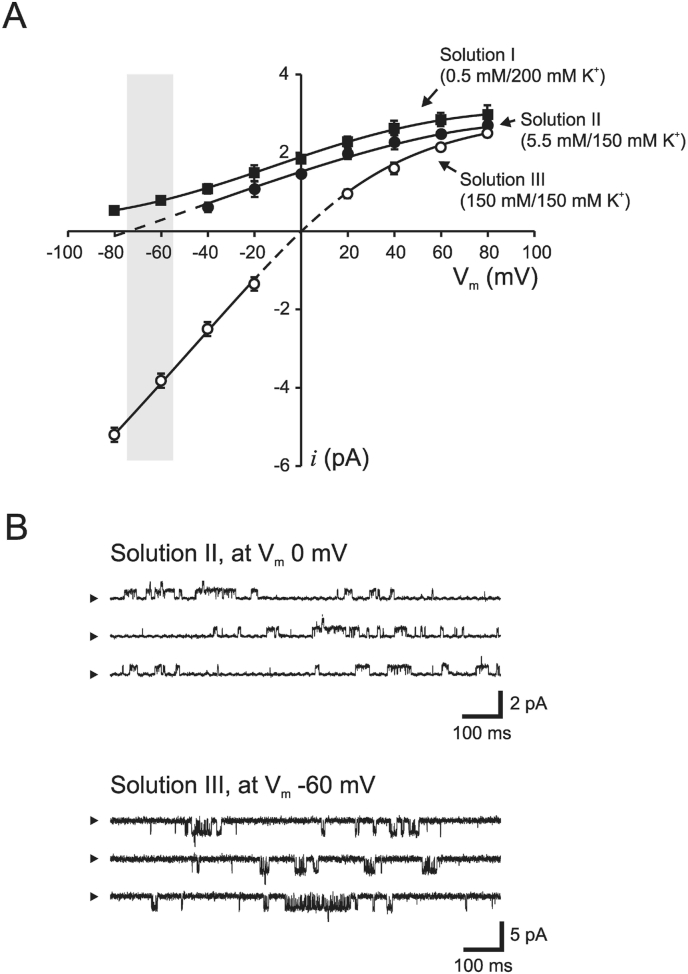
Single-channel current–voltage relationships and kinetic profiles for the K(ATP) channel. A, The current-voltage relationship for the K(ATP) channel at different K^+^ gradients was estimated using inside-out patches isolated from pancreatic β-cells. At physiological concentrations of K^+^ (solution II, closed circles), 5 mM in the pipette (outside) and 155 mM in the bath (intracellular), the reversal potential was estimated to be −78.1 ± 3.4 mV (*n* = 5). The reversal potential was right-shifted close to 0 mV, with symmetrical K^+^ concentrations (150 mM on both sides of the plasma membrane, solution III; open circles, *n* = 4). Increasing the K^+^ gradient using solution I (closed squares, *n* = 5) results in a left shift of the current-voltage relationship. The gray area indicates resting membrane potential recorded in intact β-cells at low glucose (3 mM). B, Representative single-channel recordings from inside-out patches at 0 mV (solution II) and −60 mV (solution III).

**Fig. 2:**
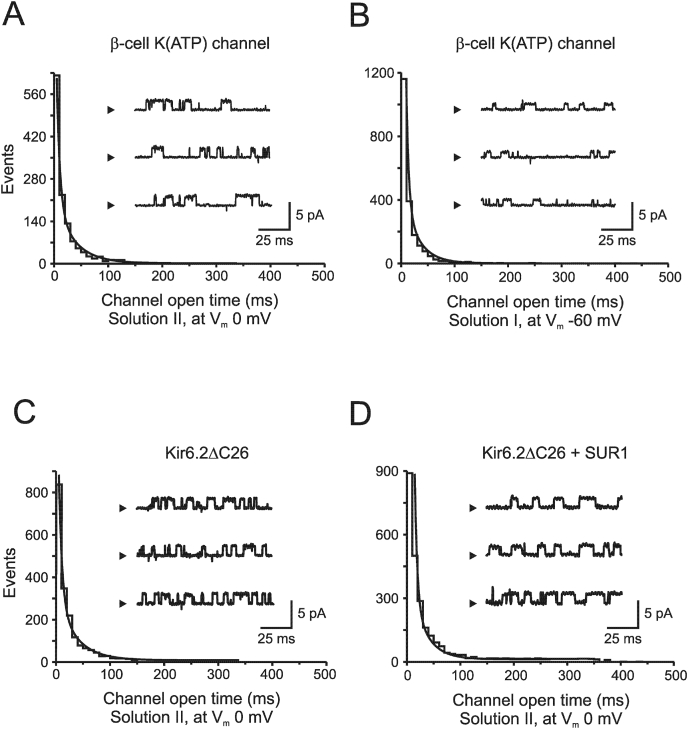
**K(ATP) channel kinetics for outward currents at different membrane potentials.** Single-channel currents recorded from an inside-out patch isolated from a pancreatic β-cell. A, At physiological ionic solution (solution II) and a membrane potential of 0 mV, channel current flow is outward and single-channel openings were described with time constants τ_o1_ = 4.9 ms and τ_o2_ = 32.1 ms, where 73% belonged to the slow component of a total of 4728 events (*n* = 4). B, At −60 mV with 0.5 mM K^+^ in the pipette (solution I), channel current flow is still outward since 45 mM KCl was also added to bath solution resulting in a total of 200 mM K^+^. The distribution of channel openings was best described by a two-exponential function with τ_o1_ = 3.7 ms and τ_o2_ = 28.8 ms (70%). A total number of 3767 events were analyzed from four different patches (*n* = 4). C-D, Single-channel currents recorded from an inside-out patch isolated from *Xenopus* oocyte injected with RNA encoding Kir6.2ΔC26 and Kir6.2ΔC26 + SUR1. All recordings were made in physiological ionic solution (solution II) and a membrane potential set to 0 mV.

**Fig. 3:**
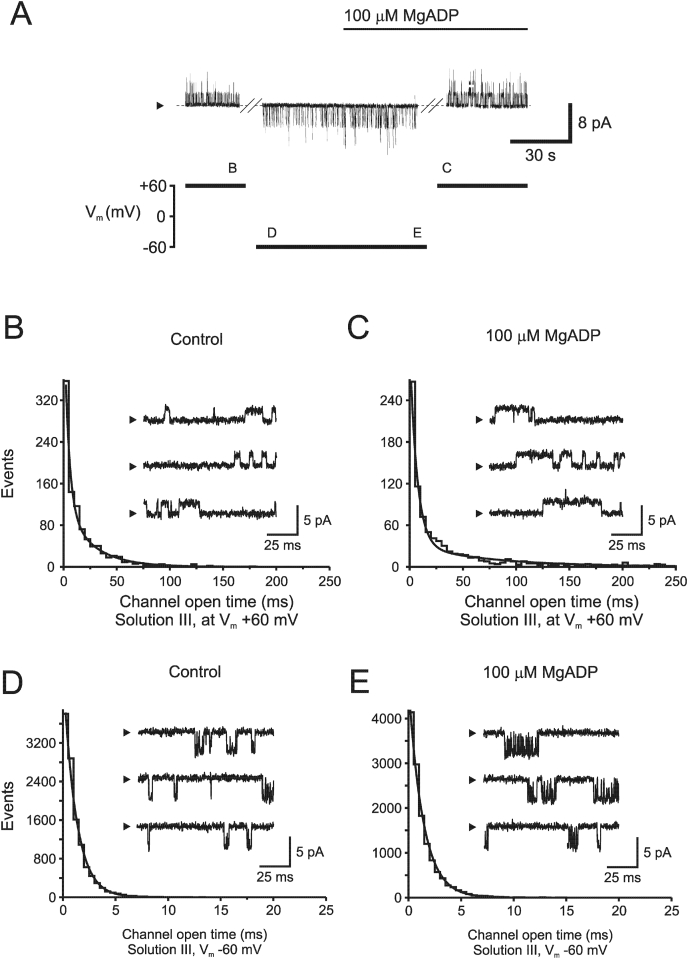
**Kinetic characteristics of outward and inward currents in the absence and presence of MgADP.** A, Channel recording (top trace) following a voltage protocol (bottom trace) used to study inward and outward kinetic events. A symmetrical K^+^ gradient was used (solution III), and the patch was excised in nucleotide-free solution at +60 mV. The membrane potential was shifted from −60 mV as indicated, and letters B, C, D, and E in the voltage-protocol corresponds to section analyzed. B, In the absence of MgADP, single channel openings were best fitted with a two-exponential distribution with τ_o1_ of 4.4 and τ_o2_ of 25.9 ms for outward currents, respectively. An addition of 0.1 mM MgADP decreased the number of slow events from 64% to 54%, but increased τ_o2_ to 72.8 ms. The fast component was not significantly altered (τ_o1_ = 6.7 ms). D, Under control conditions at −60 mV, 11,397 channel open events could be best described by a single exponential function with a τ = 1.2 ms and a mean open time = 1.2 ms. E, In the presence of MgADP (0.1 mM) channel open time was 1.3 ms (τ_o_ = 1.4 ms). A total number of 13,069 events were analyzed in the presence of MgADP (*n* = 4). Arrowhead indicates current level when the channel is closed.

**Fig. 4:**
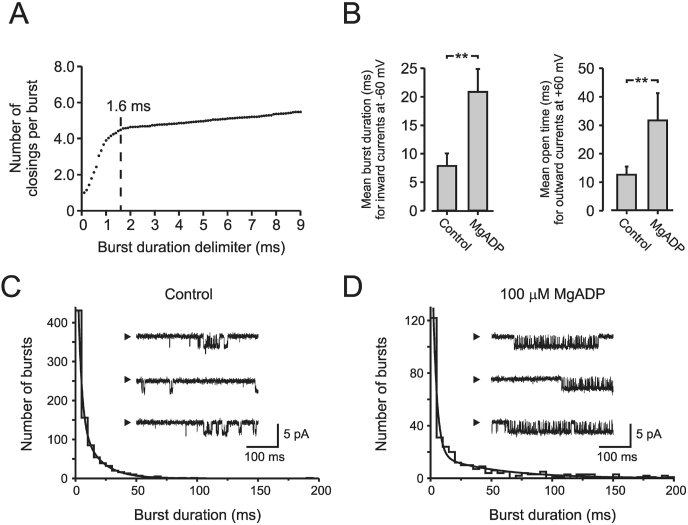
Comparison between burst duration for inward currents and channel open time for outward currents. A, Burst length delimiter *versus* number of closings per burst. The optimal burst length delimiter was estimated to be 1.6 ms (τ_cutoff_). B, Burst length for inward currents increased significantly after inclusion of 0.1 mM MgADP in the bath solution, in analogy with channel open time for outward currents. All recordings in B were made in symmetrical K^+^ concentration (solution III), and V_m_ −60 mV for inward currents and +60 mV for outward current. C and D, using a τ_cutoff_ of 1.6 ms, burst duration for inward current was plotted in the absence (C) and presence of MgADP (D). A total number of 4302 bursts were analyzed. *n* = 4–6, **P < 0.01.

## 3 Results

### 3.1 Current-voltage relationship and single-channel characteristics at different K^+^ gradients

At physiological K^+^ gradients ([K^+^]_o_ ∼5 mM and [K^+^]_i_ ∼155 mM) K(ATP) channel currents flow outward at membrane potentials more positive than the equilibrium potential for K^+^ (Fig. 1A, *closed circles*). At low glucose (<5 mM), the β-cell is polarized to approximately −70 mV [[18], [20]], thus ion flux through the K(ATP) channel is directed outward. However, it is clear from Fig. 1A (*closed circles*) that, in the presence of normal physiological ionic gradients and at a negative membrane potential close to the resting membrane potential (*gray area*), the currents flowing through the K(ATP) channel were small (<0.2 pA) and did not allow precise estimation of single-channel kinetics. Studies of K(ATP) channel activity close to the resting membrane potentials was enabled by applying symmetrical K^+^ gradients (∼150 mM K^+^ on both sides of the membrane, solution III). This configuration allowed monitoring of channel activity in both the cell-attached and inside-out mode at physiological relevant negative membrane potentials (Fig. 1A, *open circles*). However, in this recording mode, current flux through the channel was directed inward, which is not physiological since the current in intact cells is directed outward. Examples of single-channel recordings with these different configurations are shown in Fig. 1B.

### 3.2 K(ATP) channel kinetics for outward currents at different membrane potentials

Single-channel kinetics were analyzed for outward currents at different membrane potentials (Fig. 2). As stated above, in physiological ionic solutions (solution II) and at resting membrane potential for the pancreatic β-cell, K(ATP) channel events cannot be recorded due to low channel amplitude and signal/noise ratio. To circumvent this, we used solution I (0.5 mM K^+^ in the extracellular (pipette) solution, and 200 mM K^+^ in the intracellular solution). This configuration allowed recordings of K(ATP) channel events at −60 mV, representing outward currents. Detailed analyses showed no significant differences between mean open time for outward currents recorded at 0 mV and at −60 mV (17.9 ± 9.1 ms and 25.6 ± 5.0 ms, respectively). Similar results were observed for channel closed times, with τ_c1_ = 1.25 ms and τ_c2_ = 51.6 ms at 0 mV, and τ_c1_ = 1.28 ms and τ_c2_ = 65.9 ms at −60 mV. From these experiments we conclude that K(ATP) channel kinetics for outward currents can be described by the same number of kinetic constants regardless of membrane potential, two opened (τ_o1 and_ τ_o2_) and two closed states (τ_c1 and_ τ_c2_). A summary of kinetic constants is presented in Supplemental Table S1.

### 3.3 Single-channel kinetics for cloned K(ATP) channels

Outward currents were analyzed for Kir6.2ΔC26 channel alone and co-expressed together with SUR1 (Fig. 2C–D). Kir6.2ΔC26 channel openings were described with time constants τ_o1_ = 5.1 ms and τ_o2_ = 30.3 ms, where 63% belonged to the slow component (Fig. 2C, in total 5102 events, *n* = 5). Channel closings were best fitted by a two-exponential distribution with τ_c1_ = 1.32 ms and τ_c2_ = 55.0 ms (Fig. S1). The distribution of Kir6.2ΔC26+SUR1 openings (Fig. 2D) was best described by τ_o1_ = 4.8 ms and τ_o2_ = 38.3 ms (63%), and closings by τ_c1_ = 1.39 ms and τ_c2_ = 72.1 ms (78% belonging to the slow component). A total number of 4396 events were analyzed from 3 patch recordings (*n* = 3). Summarized in Table S1.

### 3.4 Kinetic responses to modulators

To compare the kinetics for outward and inward currents to known modulators, we used MgADP as a model compound and a protocol described in Fig. 3A. For outward currents, addition of 100 μM MgADP increased the time in open state and the single-channel distribution was best fitted to a two-exponential function, where the fast component remained relatively unchanged compared to the control situation (Fig. 3B–C). The slow component increased significantly to 72.8 ms (*n* = 6; P < 0.01). Subsequently, we examined the effect of MgADP on channel kinetics for inward currents using the same current trace (Fig. 3A). In the control situation (Fig. 3D), mean open time was estimated to be 1.17 ± 0.4 ms and 1.34 ± 0.5 ms after inclusion of MgADP (*n.s.*). Open time distributions were, in the absence and presence of MgADP, best approximated by single exponentials. The open time constants (τ_o_) were found to be 1.2 ms and 1.4 ms, respectively. In the absence and presence of MgADP, channel closures were fitted to two exponentials. Channel closed times showed a two-exponential relation with τ_c1_ = 0.61 ms and τ_c2_ = 55.7 ms. The presence of 0.1 mM MgADP decreased the number of events belonging to the slow component from 28% to 17%, and changed τ_c1_ to 0.52 ms and τ_c2_ to 60.4 ms (Fig. S2). Channel closures are summarized in Table S2.

### 3.5 Burst kinetics for inward currents

As shown above for outward currents, both channel openings and channel closings were best described by a two exponential function. No fast closings within channel openings were observed. The fast closings, often referred to as ‘flicker block’, occurring during inward currents are not present during outward currents and are not affected by known modulators of the K(ATP) channel [8,22]. We therefore analyzed burst kinetics for inward currents, omitting the fast closures. For inward currents, τ_cutoff_ was determined to be 1.6 ms (Fig. 4A). At τ_cutoff_ of 1.6 ms, the number of events per burst will not increase following a further increase of the burst length delimiter. Previous reports have estimated similar values of τ_cutoff_, ranging from 0.6 to 3 ms [4,17,23]. Considering bursts as long openings interrupted with fast closures, the burst time histogram was best fitted to a two-exponential distribution with time constants of 3.4 and 25.9 ms (Fig. 4C). The intraburst interval (i.e. channel closed time) was also significantly better fitted to a two-exponential distribution, compared to a single exponential (not shown). Addition of MgADP resulted in a burst duration distribution with time constants of 4.1 and 67.0 ms (Fig. 4D). Both burst duration and intraburst interval (in the presence and absence of MgADP) were significantly better fitted with two components, compared to one. The fitting was not improved exploring a three-state model.

The average burst duration for inward currents and channel open time for outward currents were increased by nearly 3-fold in the presence of MgADP (Fig. 4B). For inward currents, the short closings within bursts changed less than 11% (0.61–0.53 ms, *n.s.*), and openings less than 15% (1.16–1.34 ms, *n.s.*). The number of openings per burst increased from 3.9 ± 1.0 to 9.1 ± 1.5 in the presence of MgADP (*n* = 3; P < 0.01).

## 4 Discussion

The present study focuses on the kinetic behavior of the K(ATP) channel under normal physiological conditions. The kinetic properties of the channel under these conditions are not clear since channel recordings at physiological ionic gradients and at a negative membrane potential, close to the resting membrane potential of the β-cell (approximately −70 mV), result in low single-channel conductance. Various aspects of the kinetic properties of the K(ATP) channel, both native and reconstructed, have been extensively investigated, but mostly for inward currents [[6], [7], [8],[17], [18], [19],^25^,^26^], whereas only a limited amount of work has been focused on channel kinetics for outward currents [9,10]. Several reports have shown that the burst kinetics, similar to those seen in Fig. 1, only occur with inward currents, whereas outward currents have openings, which are considerably longer and not grouped in bursts [9,18,21,22]. However, these fast closures for inward currents, often referred to as ‘flicker’ block, likely also exist for outward currents but are much faster compared to inward currents and cannot be completely resolved even at high sample rates up to 100 kHz [27]. Large portions of these events are probably missed in our recording mode since we filter our recordings at 2 kHz and sample at 10 kHz, and only randomly occurring events are detected. However, at low K^+^ flux at physiological ionic gradients as for outward currents, the flickery block of the K(ATP) channel becomes small [24].

Using a high K^+^ gradient, corresponding to 200 mM intracellularly and 0.5 mM extracellularly (solution I), single-channel recordings are achievable close to the resting membrane potential of the β-cell (Fig. 2). In Fig. 2 outward current kinetic parameters were estimated at two different membrane potentials, 0 and −60 mV. In both these experimental setups, two opened and two closed states are required to describe K(ATP) channel kinetics for outward currents. The single-channel kinetic properties are likely confined to the Kir6.2. In our experiments no differences in the number of kinetic states could be seen for single-channels between Kir6.2ΔC26 alone and Kir6.2ΔC26 + SUR1 for outward currents (Fig. 2), which fits with observation that pore opening is associated with coordinated structural changes within the channel gate in Kir [28]. However, the time constants and transition rates are influenced by SUR1 since ATP, ADP, and other modulators, interacts with SUR1 [[29], [30], [31], [32]]. In isolated patches, several modulators are known to prolong the open state of the channel for outward currents, such as MgADP [8], diazoxide [10], and acyl-CoA ester [23].

Considering the data presented in Fig. 4, it is clear that MgADP increases channel activity by lengthening the burst duration for inward currents, i.e. increasing the number of openings per burst, and shortening the closed intervals between bursts. Open and close times within the burst, intraburst openings and closings, are unaffected by ADP. This is in good agreement with previously published findings [[29], [30], [31]]. For outward currents, however, where no bursts are observed, MgADP activates the K(ATP) channel by increasing the open time and reducing the closed time also in agreement with previously published studies [10]. ADP increases the mean open time for outward currents by 3-fold, which is analogous with the burst duration for inward currents (Fig. 4B).

In summary, we conclude that outward K(ATP) channel currents are not grouped in bursts regardless of membrane potential, and channel open time for outward currents corresponds to burst duration for inward currents.

## Grant support

This work was supported by grants from the 10.13039/501100004359Swedish Research Council, The Skandia Insurance Company Ltd, the Swedish Diabetes association, Strategic research program in Diabetes at 10.13039/501100004047Karolinska Institutet, the Berth von Kantzow's Foundation, the ERC-2018-AdG 834860 EYELETS, Diabetes Wellness Foundation, Stitching af 10.13039/100009604Jochnick Foundation, The Family 10.13039/501100004063Knut and Alice Wallenberg Foundation, the 10.13039/501100009708Novo Nordisk Foundation, Funds at 10.13039/501100004047Karolinska Institutet, the 10.13039/100010823Tore Nilsson Foundation, the Söderberg Foundation, the Thuring Foundation, the 10.13039/100008738Jeansson Foundations, the Åke Wiberg Foundation, Magn. Bergwall Foundations, the Stockholm County Council, the Family Erling-Persson Foundation, and the National Institute of Biomedical Imaging and Bioengineering of the National Institutes of Health (R01EB007047).

## Contribution statement

All authors added significant contribution in all parts of the study, including study design, interpretation of the data, and conclusions. RB and OL have performed patch-clamp recordings, and IL and BL performed the molecular biology procedures. All authors read and approved the final version of the manuscript.

## Declaration of competing interest

The authors declare that they have no known competing financial interests or personal relationships that could have appeared to influence the work reported in this paper.

## Figures and Tables

**figs1:**
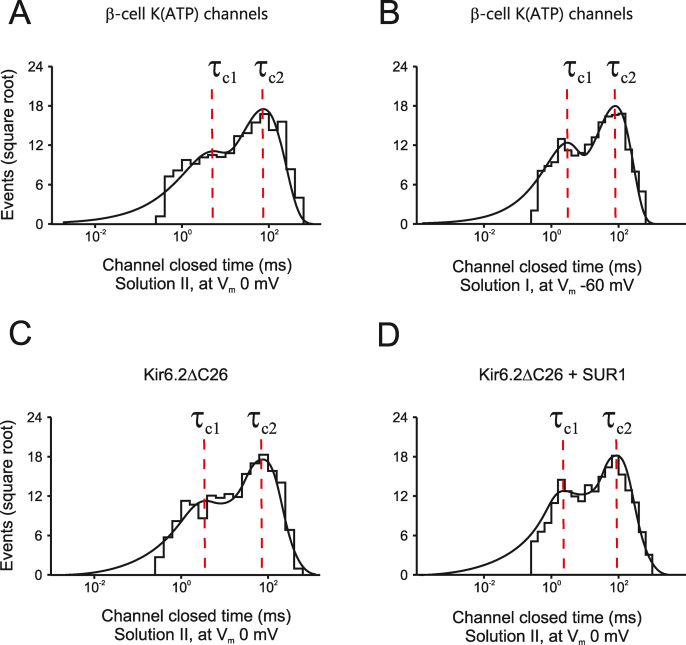


**figs2:**