# Supplementary material for: Inward and outward currents of native and cloned K(ATP) channels (Kir6.2/SUR1) share single-channel kinetic properties
Source: Biochem Biophys Rep. 2022 Apr 8;30:101260. doi: 10.1016/j.bbrep.2022.101260 (PMC9006676; doi:10.1016/j.bbrep.2022.101260)
Supplement: Multimedia component 1 [file mmc1.pdf]

Table S1

**Outward currents at**	0 mV						-60 mV					
	τ_{o1}	τ_{o2}	% slow component	τ_{c1}	τ_{c2}	% slow component	τ_{o1}	τ_{o2}	% slow component	τ_{c1}	τ_{c2}	% slow component
β-cell K(ATP) channels	4.9	32.1	73%	1.3	51.6	66%	3.7	28.8	70%	1.28	65.9	62%
Kir6.2ΔC26	5.1	30.3	63%	1.3	55.0	72%						
Kir6.2ΔC26 + SUR1	4.8	38.3	63%	1.4	72.1	78%						
