# Supplementary material for: Inward and outward currents of native and cloned K(ATP) channels (Kir6.2/SUR1) share single-channel kinetic properties
Source: Biochem Biophys Rep. 2022 Apr 8;30:101260. doi: 10.1016/j.bbrep.2022.101260 (PMC9006676; doi:10.1016/j.bbrep.2022.101260)
Supplement: Multimedia component 2 [file mmc2.pdf]

Table S2

**β-cell K(ATP) channels**	Control conditions						0.1 mM ADP					
	τ_{o1}	τ_{o2}	% slow component	τ_{c1}	τ_{c2}	% slow component	τ_{o1}	τ_{o2}	% slow component	τ_{c1}	τ_{c2}	% slow component
Outward currents at +60 mV	4.4	25.9	64%	3.4	92.7	78%	6.7	72.8	54%	2.9	95.2	72%
Inward currents at -60 mV	1.2	-	-	0.61	55.7	28%	1.4	-	-	0.52	60.4	17%
